# A generalized mathematical framework for estimating the residue function for arbitrary vascular networks

**DOI:** 10.1098/rsfs.2012.0078

**Published:** 2013-04-06

**Authors:** Chang Sub Park, Stephen J. Payne

**Affiliations:** Department of Engineering Science, Institute of Biomedical Engineering, University of Oxford, Parks Road, Oxford OX1 3PJ, UK

**Keywords:** residue function, flow heterogeneity, ischaemic stroke

## Abstract

The microvasculature plays a vital part in the cardiovascular system. Any impairment to its function can lead to significant pathophysiological effects, particularly in organs such as the brain where there is a very tight coupling between structure and function. However, it is extremely difficult to quantify the health of the microvasculature *in vivo*, other than by assessing perfusion, using techniques such as arterial spin labelling. Recent work has suggested that the flow distribution within a voxel could also be a valuable measure. This can also be measured clinically, but as yet has not been related to the properties of the microvasculature due to the difficulties in modelling and characterizing these strongly inter-connected networks. In this paper, we present a new technique for characterizing an existing physiologically accurate model of the cerebral microvasculature in terms of its residue function. A new analytical mathematical framework for calculation of the residue function, based on the mass transport equation, of any arbitrary network is presented together with results from simulations. We then present a method for characterizing this function, which can be directly related to clinical data, and show how the resulting parameters are affected under conditions of both reduced perfusion and reduced network density. It is found that the residue function parameters are affected in different ways by these two effects, opening up the possibility of using such parameters, when acquired from clinical data, to infer information about both the network properties and the perfusion distribution. These results open up the possibility of obtaining valuable clinical information about the health of the microvasculature *in vivo*, providing additional tools to clinicians working in cerebrovascular diseases, such as stroke and dementia.

## 1. Introduction

Cerebrovascular disease is one of the most significant problems in clinical medicine. With an increasingly ageing population, the incidence of these diseases, including stroke and vascular dementia, is set to rise significantly [1]. Cerebral perfusion plays a critical role as the supply of blood to regions of tissue must be maintained at a continuous level at all times, with even short interruptions to this supply leading potentially to localized regions of tissue damage and death. The cerebral microvasculature thus plays a vital role in the matching of demand and supply of nutrients to regions of brain tissue. Clinical measurements of perfusion are very widely used. Several techniques are available to measure perfusion, including computed ultrasound and magnetic resonance imaging (MRI). Two common MRI techniques for the quantification of cerebral perfusion are dynamic susceptibility contrast MRI and arterial spin labelling, which are widely used as research tools. These techniques yield important information such as cerebral blood flow (CBF), vascular mean transit time (MTT) and flow heterogeneity. CBF and MTT are well-established tracer-kinetic parameters that are independent of the imaging technique employed; they are thus of particular interest, since they can be acquired in a variety of modalities. CBF has been used to delineate the penumbra, tissue at risk but still viable, in ischaemic stroke patients; the threshold values from the different perfusion imaging studies, however, vary significantly and this variability raises the question of accuracy [2]. MTT is commonly used to interrogate the efficacy of flow delivery and often provides complementary information to CBF. Most recently, flow heterogeneity has been investigated and related to cerebral metabolism, with the hypothesis that the degree of heterogeneity, owing to the multiple capillary pathways, may play a major role in brain function [3].

**Figure 1.**
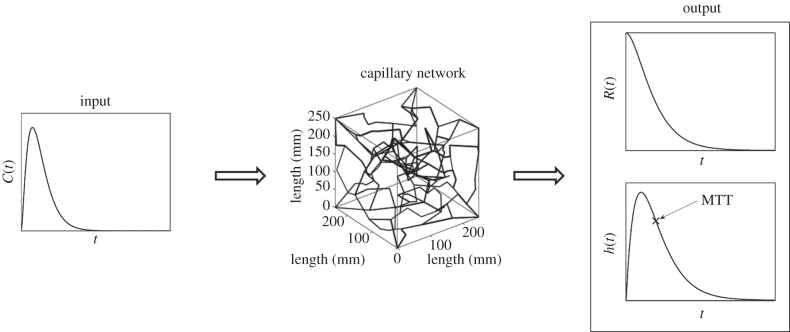
Block diagram of the model overview.

Accurate measurements of the above quantities are desired to improve our understanding of ischaemic stroke. Current techniques to obtain CBF, MTT, residue function and flow heterogeneity are mainly based on deconvolving the arterial input function (AIF) with the concentration of the tracer in the volume of interest [3–5], measured using MRI, although this information can be obtained from other imaging modalities. Figure 1 shows a model overview with an AIF into the capillary network and sample plots of some of the results that can be obtained, such as residue function and transit time distribution.

**Figure 2.**
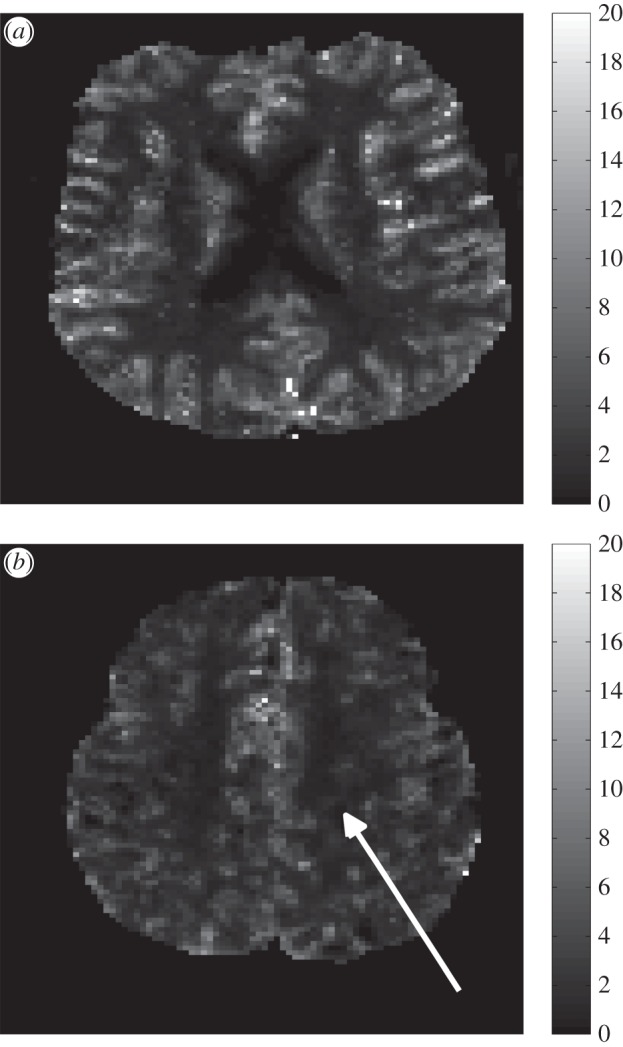
CBF map, using arbitrary units, of a healthy subject (*a*) and an ischaemic stroke patient (*b*) taken from Mehndiratta *et al.* [6]. The white arrow in (*b*) represents the infarct region.

There are two deconvolving methods, the transform approach and the algebraic approach. The former considers a Laplace or Fourier transform while the latter is based on discretizing the variables leading to a matrix problem. The discretizing approach is generally an ill-posed problem requiring the use of singular value decomposition (SVD) to estimate the impulse response, where the impulse response is proportional to the residue function, the fraction of tracer present in the capillary network at a specific time. SVD, as well as the variations of the SVD approach, however, leads to underestimation of CBF as well as oscillations in the residue function which makes estimation of related properties difficult. A vascular model composed of a major artery feeding arterioles in parallel was proposed to improve the limitations of solely using the SVD method [3,4]: however, this relies on a pre-defined variability in the pathway transit times. Recently, a novel convolution algorithm was presented by Mehndiratta *et al.* [6] where the residue function is presented by control points, each with a certain degree of freedom, followed by a cubic spline interpolation through these control points. This method has been shown to improve the accuracy of the estimated CBF when compared with the actual CBF. Figure 2 shows a sample CBF image, plotted in arbitrary units, of a healthy subject and an ischaemic stroke patient obtained from Mehndiratta *et al.* [6].

The recent improvements in estimating residue function opens up the possibility of relating this function to the underlying capillary network properties. The work by Jespersen & Østergaard [7] shows how the transit time distribution is directly related to the residue function; however, they considered a non-physiological network of parallel pathways with different transit times set by an underlying distribution. Using this approach, it is not possible to relate changes in the residue function to the properties of the network. In order to compare results obtained from mathematical models with *in vivo* results, a physiologically accurate model is required. Many vascular network models are available in the literature ranging from cerebrovascular networks created using a specific algorithm [8–10] to accurate arterial network models consisting of only the main arteries [11]. The flow model considered in these studies depends mainly on the size of the vessels considered. A one-dimensional area-averaged flow model is considered for arteries with wall deformations while a Poiseuille flow model is considered for capillaries where wall deformations are negligible. Other recent work [12] has shown how physiologically accurate capillary networks that match experimental data can be generated computationally. In this paper, we unite these two approaches by simulating the residue function in physiologically accurate capillary networks and investigating changes in the resulting residue function under different conditions. We also propose a new multi-scale mathematical framework for estimating the residue function for any arbitrary network and a new characterization of this function that can be used both for model simulations and clinical data. This approach thus enables us to show how additional clinical information can be extracted from existing perfusion measurements, which has significant potential clinical value.

## 2. Theory

### 2.1. Single-vessel residue function

The residue function, *R*(*t*), can be obtained via the following equation where the concentration of the tracer in the capillaries, 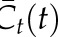, is proportional to the convolution of the concentration at the input, 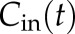, with *R*(*t*)2.1
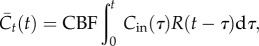
where CBF is the constant of proportionality measured in per unit second. To obtain the concentration in the capillaries, the mass transport equation is solved for each vessel; the one-dimensional mass transport equation can be expressed as2.2
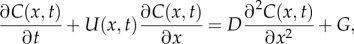
where *C* is the concentration, *x* is the axial coordinate, *U* is the axial velocity of blood, *D* is the diffusion coefficient and *G* is a generation term. In order to simplify the problem at hand, a passive transport is considered, hence the tracer stays within the vessel and thus there is no generation of the tracer, with the diffusion term ignored since the convective term is much more significant and a Poiseuille flow is assumed. These assumptions will be examined later. Taking the Laplace transform of both the residue function equation for a single vessel and the convection driven one-dimensional mass transport equation and taking into consideration that *C*(*x*,0) = 0 gives2.3
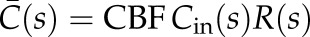
and2.4
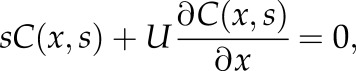
respectively. From the latter equation, the concentration in the *s*-domain can thus be expressed as2.5
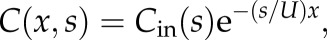
where *C*_in_(*s*) is the concentration at the inlet of the vessel. The concentration at the outlet, 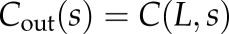. Thus, the transfer function, *X*(*s*) can be expressed as2.6
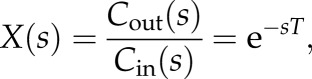
where *T = L/U* is the time taken to travel from the inlet to the outlet. The volume-averaged concentration in the vessel can then be obtained from2.7
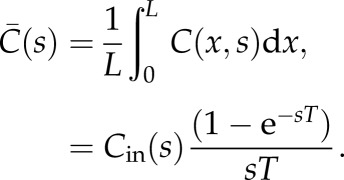
The residue function in the *s*-domain can thus be expressed by combining equations (2.3) and (2.7) as2.8
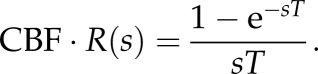
To separate CBF and *R*(*s*), it is conventionally assumed that *R*(*t* = 0) = 1. Using the initial value theorem, this can be expressed as2.9
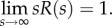
Thus,2.10
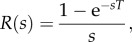
which converted back to the time domain can be represented as2.11
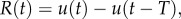
where *u* is a unit step. Hence *R*(*t*) is a simple rectangular function with width equal to the transit time of the vessel.

The transit time distribution, *h*(*t*), can then be obtained directly from *R*(*t*) [3] using2.12
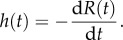


The transit time in *s*-domain can be expressed as *h*(*s*) = exp(–*sT*) and in the time domain as2.13
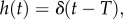
where *δ* is the Kronecker delta. Having obtained the residue function and transit time distribution for a single vessel we now proceed to consider these for an arbitrary network of vessels.

### 2.2. Network residue function

The concentration of a tracer in a network, 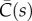, can be solved by taking the sum of the product of the individual vessel average concentration and volume and dividing this by the total vessel volume2.14
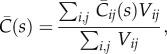
where the subscript *ij* refers to a vessel connected between nodes *i* and *j*, *V*_*ij*_ is the volume and 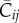 is the average concentration in vessel *ij*. If nodes *i* and *j* are connected, then both the volume and average concentration will have a value assigned, otherwise they will be zero. To solve for the vessel concentrations in the network the concentration at the nodes need to be known to set the inlet concentration for each vessel. This is calculated for each node *j* from a flow rate weighted sum of all supply vessels2.15
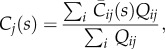
where *C*_*j*_ is the concentration at nodes *j* and *Q*_*ij*_ is the flow from nodes *i* to nodes *j*, where only nodes *i* will be considered if nodes *i* and *j* are connected. The ratio of the average concentration in a vessel to the input can thus be expressed as2.16
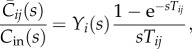
where2.17
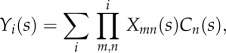
where *X*_*mn*_(*s*) is the transfer function for any vessel connected between nodes *m* and *n* defined in equation (2.6). *Y*_*i*_(*s*) then considers all the possible pathways from the input to the inlet node, *i*, of the vessel being considered. Substituting equation (2.16) into equation (2.14), and since *Q = V/T*, the ratio of the averaged concentration in the vessel network, 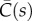, to the input, i.e. the tissue response function, can be expressed as2.18
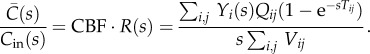
Using the initial value theorem again yields2.19
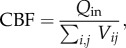
where *Q*_*in*_ is the flow at the input. The residue function can thus finally be expressed as2.20
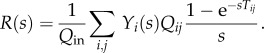


Taking the inverse Laplace transform, the residue function in the time domain can thus be expressed as2.21
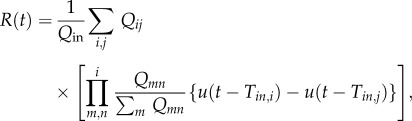
where *T*_*in,i*_ and *T*_*in,j*_ are the transit times from the input to nodes *i* and *j*, respectively. Equation (2.21) represents the sum of contributions from all vessels, each of which is a product of all pathways to it, and hence the residue function is a sum of rectangular functions characterized by a width, a time delay and a magnitude. The width is dependent on the transit time of the vessel being considered, whereas the time delay is dependent on the transit time from the input of the network to the inlet of the vessel and the magnitude is dependent on the product of the inflow ratios at each node that it has passed through from the inlet of the network to the inlet of the vessel. It is thus only necessary to know all the possible pathways from the input to each node within the network as well as the flow in each vessel. Equation (2.21) then provides an explicit expression enabling the residue function to be calculated knowing only the vessel flows and transit times, and the pathways to each vessel.

### 2.3. Capillary network residue function

We now consider a physiological network, that of capillary vessels in the human brain. It would clearly be ideal to generate microvascular structures from real brain tissue and to run simulations on such networks. This process, however, is extremely complex and time consuming as it involves sample preparation, scanning and image processing as well as being very difficult to do *in vivo*. Thus, a previously developed network creation algorithm [12] is considered here to generate artificial networks matching statistical data obtained from human cerebral tissue samples [13] and to solve for the flow fields in these networks. An advantage of this model is that statistical variations in network properties can be investigated in terms of their effects on the residue functions.

**Figure 3.**
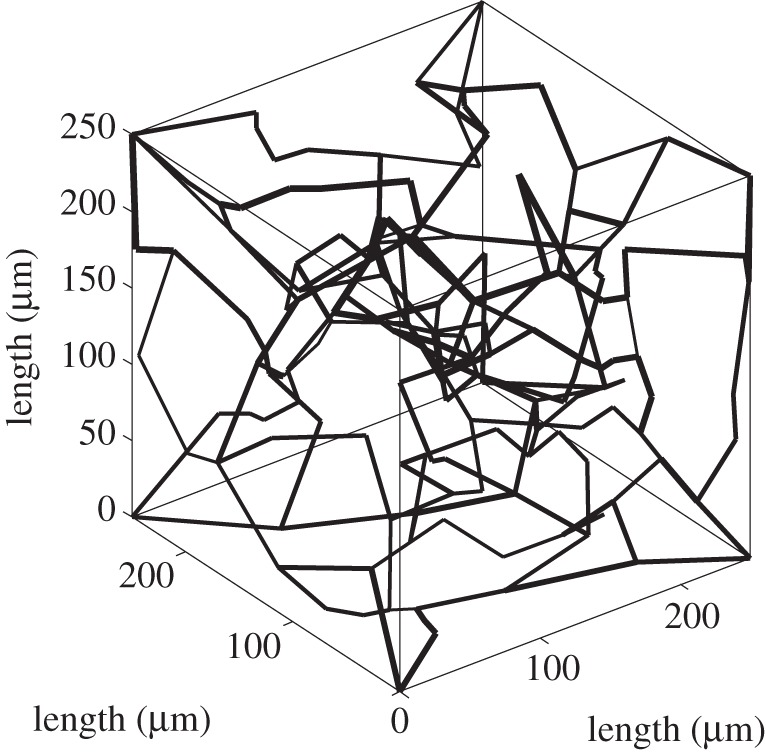
Plot of a randomly created network.

The approach proposed by Su *et al.* [12] uses Prim's algorithm to obtain a minimum spanning tree by connecting nodes created at random coordinates in a cube. Vessels of specific lengths are then added and removed to match the length distribution of the capillaries with experimental data obtained by Cassot *et al.* [13] and the radius of the vessels likewise. Once the network is created, the flow is solved as outlined in Su *et al.* [12]. This flow model is based on a Poiseuille flow with individual vessel viscosity calculated based on vessel diameter and assuming a constant haematocrit of 0.45, using the relationship proposed by Pries *et al.* [14]. The resulting equations are written in matrix form and solved. A constant haematocrit is assumed here to simplify the flow model: this assumption will be relaxed in future work. For full details of the networks analysed here, the reader is referred to Su *et al.* [12], where all such information is given. Figure 3 shows a sample capillary network created using this algorithm.

### 2.4. Characterization

Some kind of distribution is required to characterize the solution. Four conditions must be satisfied by any chosen function, these being the residue starting at one and decaying towards zero with time and the transit time starting at zero and heading towards zero as time goes to infinity. Several studies have considered a single gamma distribution to model *h*(*t*), for example [3,4,7], such that2.22
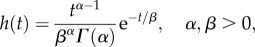
where *α* and *β* are parameters that define the distribution. The mean of this distribution, *μ* = *αβ*, thus represents MTT by definition. The corresponding variance, 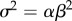. The residue function in the *s*-domain becomes2.23
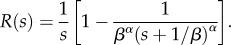


The gamma distribution satisfies all the boundary conditions previously stated as long as *α* > 1. This can be extended to provide a more general distribution for *h*(*t*) as a sum of gamma distributions:2.24
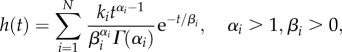
and hence2.25
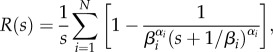
where *k*_*i*_ is the weighting of each gamma distribution. In order to satisfy the boundary conditions 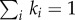 and 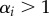.

## 3. Results

The initial conditions to solve for the flow are set such that the pressure difference between the arteriole–capillary and capillary–venule junctions is 1000 Pa. This value was chosen so that the average CBF for the capillary networks considered here was around 50–60 ml/100 ml min^−1^, which is a common range of values obtained in a healthy human brain [15]. The network cube has an edge length of 250 µm and an arteriole–capillary and capillary–venule junction density matching that obtained from experimental results [16], in this case three and two, respectively. This method will be applied to 20 different networks with equivalent conditions, thus allowing statistical analysis to be performed, followed by varying the flow and network conditions to quantify the effects of such changes on the residue function. Table 1 shows details of the morphology of the created networks averaged over the 20 networks. These values are compared with experimental results (M1 and M2) obtained by Cassot *et al.* [13]. Note that changing the initial pressure difference condition keeping the same network structure changes CBF, which in turn changes the transit time. Since the ratio of the flows at the nodes are the same, the residue function plot will retain its shape but with different time scales. This time scale is linearly dependent with CBF, thus changes in pressure difference will not affect the residue function plot with a CBF normalized time scale.

**Table 1.** Mean and s.d. of morphological parameters compared with experimental data (M1 and M2).

	M1	M2	artificial network
vessel density (1/mm^3^)	8817	7219	7718
connectivity	3.11	3.16	3.22
length (µm)	57.37 ± 50.98	63.26 ± 53.73	59.71 ± 51.61
diameter (µm)	6.91 ± 3.85	5.91 ± 1.30	6.24 ± 1.30

**Figure 4.**
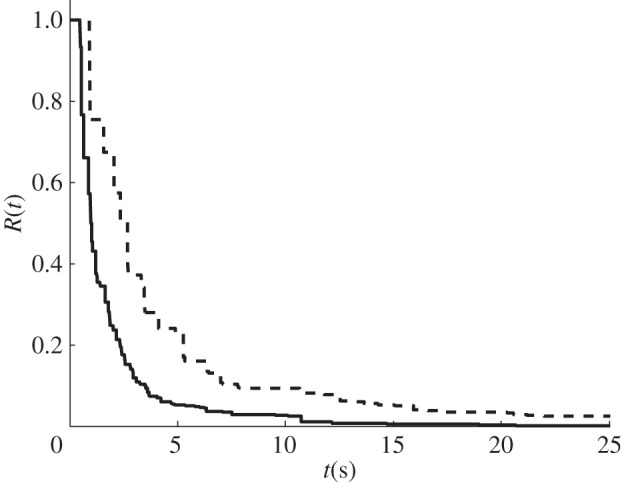
Plot of *R(t)* for a randomly chosen sample capillary network for a normal (solid line) condition and for a 20% (dashed line) blockage.

Figure 4 shows a sample plot of the residue function in our base cube for one capillary network. The residue function is composed of many rectangular functions, with a time delay from the inlet of the capillaries, and decays gradually with time due to the decrease in the magnitude of the rectangular functions downstream. A sharp decay is initially observed with a 90 per cent drop in the residue function during the first few seconds followed by a slow decay where the remaining 10 per cent drops. The fast and slow decays seem to represent the ‘shorter’ and ‘longer’ pathways available, respectively, from the arteriole–capillary junctions to the capillary–venule junctions; this will be discussed in more detail later.

The next case considered is when 20 per cent of the vessels in the capillary networks are randomly blocked. Figure 4 shows a sample plot of the residue function with the vessels blocked. Once more, a decay in the residue function is observed, but this is slower as more vessels are blocked. This is due to there being fewer pathways available and it is thus expected for the contrast agent to take longer to be flushed out of the system. A sharp initial decay is still observed, in this case a 90 per cent drop in the residue function during the first few seconds followed by a slow decay where the remaining 10 per cent drops. Note that the change from the fast decay to the slow decay is more gradual than for the normal case.

In order to compare the two conditions, a bigamma distribution is now considered to characterize the residue function. This particular distribution, which was considered for all the 20 generated networks, is chosen here due to its simplicity as well as its ability to capture the shape of the residue function. A residual sum of squares (RSS) was considered during the curve fitting process. RSS is a measure of the discrepancy between the data and the estimation model, the bigamma distribution in this case. Although a gamma and a trigamma distribution were also considered, these were discarded as the former was over constrained, leading to a relatively large RSS as shown in table 2, while the latter was found to be underconstrained, leading to difficulties in fitting the residue function.

**Table 2.** Mean and s.d. of RSS for the different cases considered.

	monogamma	bigamma
normal	3.76 ± 2.03	0.67 ± 0.31
20%	5.10 ± 4.48	1.11 ± 0.65

**Figure 5.**
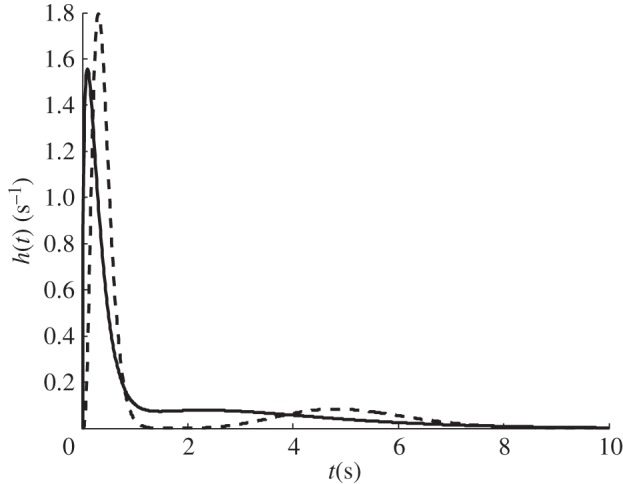
Plot of a characterized *h(t)* for a randomly chosen sample capillary network for a normal (solid line) condition and for a 20% (dashed line) blockage.

Figure 5 shows a plot of the characterized transit time distribution for a normal and 20 per cent blocked case. Owing to the nature of the distribution, there are two peaks in the transit time distribution plots. The peaks are further apart when 20 per cent of the vessels are blocked. The MTT can then be obtained from the transit time distribution or from the ratio of CBV to CBF.

**Figure 6.**
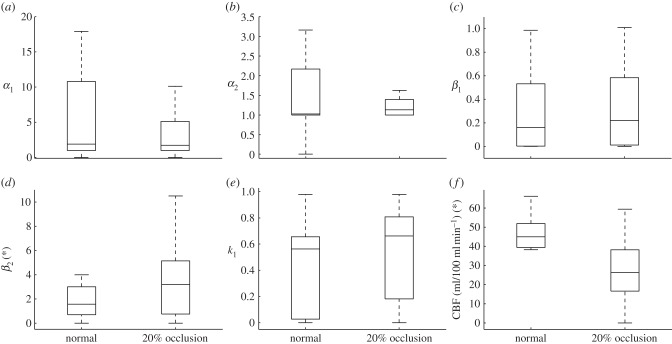
Boxplot comparing the gamma distribution parameters between the normal and 20% occluded case.

Boxplots of the different parameters are presented here in order to compare more quantitatively the normal to the ischaemic conditions. Outliers have been ignored in all of the following boxplots. Figure 6 shows the two shape parameters, *α*_*i*_, the two time constants, *β*_*i*_, the weighting, *k*_1_, and CBF for the two conditions considered here. For the normal condition, the median of the two shape parameters, 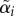, have the same order of magnitude with 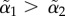. Both shape parameter boxplots are positively skewed. The median of the time constants, 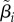, have different orders of magnitude. The smaller time constant, 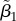, being an order of magnitude smaller than 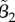. The boxplots of the time constants are also positively skewed. The boxplot of the weighting, *k*_1_, is negatively skewed and the median of the CBF being around 46 ml/100 ml min^−1^. There are no differences in the shape of the parameter boxplots between the 20 per cent occluded and normal case. There is however, a general increase in the median of the time constants as the vessels are blocked. The percentage increase in the median of the two time constant are approximately 10 per cent and 110 per cent, respectively. 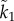 also increases by approximately 25 per cent which means that 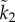 decreases. The medians of the shape parameters and CBF drop by approximately 45 per cent, 15 per cent and 40 per cent. The drop in CBF median is due to there being fewer collateral pathways, which implies an increase in the net resistance and thus for the same change in pressure from the arteriole–capillary junctions to the capillary–venule junctions, CBF decreases. The changes in the time constants and the weighting suggest that the longer pathways are more significantly affected by the blockage of the vessels. A change in pressure difference changes both time constants, but not the shape parameters.

Table 3 shows the mean and s.d. of the different parameters in the bigamma distribution for both the normal and the ischaemic cases. Note that only *k*_1_ is tabulated since *k*_2_ = 1−*k*_1_. For the normal case, the mean of the two shape parameters, 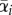, have the same order of magnitude with 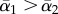. The mean of the time constants, 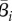, have different orders of magnitude, 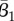 being an order of magnitude smaller than 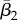. The 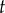-test between the two time constants suggest a significant difference in the mean since the *p*-value was less than 0.05. The mean CBF is approximately 50 ml/100 ml min^−1^. Blocking 20 per cent of the vessels leads to a drop in the average CBF by approximately 40 per cent with the weighting between the two gamma distribution shifting more towards the smaller time constant. A paired *t*-test analysis is performed on the parameter data between the normal and 20 per cent occluded case to quantify any significance in the variation with an asterisk (*) representing those parameters with a *p*-value less than 0.05. There is thus a significant difference in the mean for the parameters *β*_2_ and CBF between the two conditions.

**Table 3.** Mean and s.d. of *k*_*i*_, *α*_*i*_, *β*_*i*_ and CBF for the different cases considered.

	*k*_1_	*α*_1_	*α*_2_	*β*_1_	*β*_2_ (*)	CBF (*)
normal	0.53 ± 0.28	9.68 ± 12.08	2.29 ± 2.56	0.41 ± 0.43	2.14 ± 1.09	50.00 ± 9.46
20%	0.68 ± 0.25	10.99 ± 20.64	3.55 ± 3.95	0.44 ± 0.45	4.96 ± 4.18	32.40 ± 12.45

## 4. Discussion

It has been shown here that the residue function of a single vessel can be solved analytically as a rectangular function, the width being dependent on the transit time of the vessel. However, to solve for the residue function in the capillary network requires a computational approach due to the large number of vessels as well as the interconnection between the vessels, which makes it complex to find all the different possible pathways from the arteriole–capillary junctions to the capillary–venule junctions. Our solutions show that the residue function exhibit an initial fast decay where approximately 90 per cent of the drop can be observed in the first few seconds followed by a slow decay of the remaining 10 per cent, which seems to represent the ‘shorter’ and ‘longer’ pathways, respectively. A similar observation was made by Mehndiratta *et al.* [6], when solving for the residue function from images of a human brain using their novel control point interpolation method. This suggests that the residue function of such networks with a large number of different pathways can be characterized relatively simply by a combined short and long pathway solution. The two methods also agree in the approximate time for which the residue function decays to close to zero, this being between 10 and 20 s for the healthy case and above 20 s for the ischaemic case. Note that the residue function obtained here is closer to an exponential shape for the healthy case and to a box-car shape for the ischaemic case, thus also agreeing, at least qualitatively, with the findings previously stated by Mouridsen *et al.* [4].

As a result, the gamma distribution seems to be the most suitable function for characterization of the residue function, as it matches both experimental data and numerical simulations well and provides the flexibility to model both healthy and ischaemic behaviour. This is in agreement with previous studies [3,4,7]: we also note that it satisfies the necessary boundary conditions, which other distributions do not. We found that a two component gamma distribution works well, with a higher order series of gamma distributions providing little additional accuracy. The finding that there appear to be two characteristic pathways and associated time constants is an interesting one and one that should be investigated further under a wider range of conditions.

This two component model also shows interesting behaviour when conditions are changed. The analysis presented here shows that a change in pressure affects both time constants equally (due to the scaling of the residue function) while blocking 20 per cent of vessels only affects the larger time constant significantly. In both cases, there is a change in CBF, but the resulting residue function changes differently. Since the residue function retains its shape with changes in pressure, the shape parameter is unaffected by pressure. Twenty per cent blockage also has no significant effect on the shape parameter. The magnitude of the parameters generally increase as the number of vessels blocked increase, and thus increasing the mean values of the two gamma distributions. The fact that different conditions affect the residue function in different ways implies that changes in these conditions could be inferred from clinical data. Since perfusion is estimated independently of residue function, this means that perfusion imaging contains significantly more information than is currently used. This is potentially very significant clinically.

Additionally, the recent work by Jespersen & Østergaard *et al.* [7] has proposed that the capillary transit time heterogeneity (CTTH) defined as the s.d. of capillary transit times, could be a valuable measure of the health of the microvasculature. Even with equal net blood flows and numbers of parallel capillary paths, a homogeneous flow distribution leads to a higher oxygen extraction fraction than a heterogeneous flow distribution. Their hypothesis is thus that homogeneous flow distribution occurs during ischaemia [3,4], hence attempting to protect the tissue during limited flow conditions by maximizing oxygen delivery. However, their capillary model is not based on physiological data. The model proposed here has overcome this limitation. The mean and standard deviation of CTTH for the normal and 20 per cent blocked cases are found to be 2.35 ± 0.52 and 3.33 ± 1.19, respectively, here. Hence, blocking of the vessels increases CTTH and thus the flow becomes more heterogeneous. Although only an initial result, this is not in agreement with the proposed hypothesis. This could be for a number of reasons, in particular that there are mechanisms that locally control blood flow, such as pericytes, which could have a strong influence. Since we have only investigated a purely passive network, further investigation will be required to identify whether the hypothesis is indeed valid. One particularly interesting potential avenue for exploration will also be the role of adaptation or remodelling in microvascular networks following ischaemia.

It should be noted that several other assumptions have been made here in deriving the residue function. First, it was assumed that the haematocrit was constant throughout the capillary network, and thus viscosity was only dependent on vessel diameter. The apparent viscosity is known to vary strongly nonlinearly with haematocrit, which in turn would affect the flow in the vessels. This assumption has been made here for the purposes of simplicity, but will be relaxed in future work when a full haematocrit model will be included. Secondly, a Poiseuille flow was considered since the Navier–Stokes equation is dominated by the viscous terms, thus assuming that the velocity over time and length was constant within the vessel. This is a reasonable assumption that is widely used in such networks. Thirdly, the mass transport equation was considered to be solely driven by convection and thus the diffusion term was ignored. The Pèclet number, which is a dimensionless quantity defined as the ratio of convection to diffusion, is found at this length scale to have a magnitude of the order of 10. Introducing the diffusion term would change equation (2.4), a first-order differential equation, to a second-order differential equation, which could then be solved leading to an exponential decay function, but this will introduce a limited amount of smoothing to the residue function, which will become less important as the model is scaled up to voxel size. Fourthly, this model has a limitation in the size of the capillary network cube being studied here, owing to limitations in computational power, the computational cost rising very rapidly with increased cube size. Creating an artificial network with a volume of 1/4 mm^3^ and calculating the flow and thus CBF can be determined in the order of 1 min on a standard PC (Intel Quad Core 2.53 GHz CPU with 12 GB RAM). Obtaining the pathways from the inlet to each node in the network is more time consuming, this being in the order of minutes. The artificial networks analysed here have a volume of 1/4 mm^3^, i.e. approximately 125 vessels. A standard imaging voxel has a volume of around 5 mm^3^, which is 20^3^ times larger. There are thus around 1 million vessels (125 × 20^3^), which would require an extremely large computation cost and memory loading. Including the pathways from the inlet to each node in the network would further increase the computation cost.

Since the fundamental goal of this work, however, is clinical applications in ischaemic stroke, to make any comparisons with clinical data it will be necessary to increase the size of the cube to that of an imaging voxel. To scale up the analysis used here will require a different approach, for example, the multi-scale approach proposed by Shipley & Chapman [17]. This will be the subject of future work. An inverse analysis could then be performed such that it would be possible to determine the properties of the capillary network from data acquired by imaging. Such a model would provide potentially very valuable information and also allow us to improve our understanding of certain post stroke effects. For example, it is well known that stroke leads to changes in the physiological properties of the brain tissue, which leads to the no-reflow phenomenon [18]. Being able to predict flow after the removal of the blockage and the effects of reperfusion could provide valuable clinical information for decision-making and planning interventions. Future work will focus on this analysis and clinical translation.

## 5. Conclusion

A novel mathematical technique has been developed to solve for the residue function in a capillary network with matching physiological topology. The residue function for a single vessel can be expressed as a rectangular function, the width depending on the vessel transit time, the magnitude depending on the flow and the delay depending on the transit time from the input to the vessel. Hence only the transit time, flow and the pathways are required to solve for the residue function in any arbitrary network such as the cerebral micro-vasculature. A two-component gamma distribution is proposed here to characterize the function so that it can be directly related to clinical data. Different condition changes have shown different changes in the residue function: a change in pressure scaled the residue function with time, keeping its shape, thus affecting equally both time constants. A 20 per cent blockage on the other hand only affected the larger time constant, with both changes affecting CBF. Vessel blockage also increased CTTH, hence the flow becoming more heterogeneous with reduced perfusion, a finding that needs further investigation in the light of other work.
